# Temporal Persistence of Bromadiolone in Decomposing Bodies of Common Kestrel (*Falco tinnunculus*)

**DOI:** 10.3390/toxics8040098

**Published:** 2020-11-07

**Authors:** Irene Valverde, Silvia Espín, Pilar Gómez-Ramírez, Isabel Navas, Pablo Sánchez-Virosta, María Y. Torres-Chaparro, Pedro Jiménez, Pedro María-Mojica, Antonio J. García-Fernández

**Affiliations:** 1 Service of Toxicology and Forensic Veterinary, Faculty of Veterinary, Campus de Espinardo, University of Murcia, 30100 Murcia, Spain; irene.valverde@um.es (I.V.); pilargomez@um.es (P.G.-R.); imnr@um.es (I.N.); pablo.s.v@um.es (P.S.-V.); mariayanneth.torresc@um.es (M.Y.T.-C.); pjjm@um.es (P.J.); pmmojica@um.es (P.M.-M.); 2 Santa-Faz Wildlife Recovery Center, Consellería de Agricultura, Desarrollo Rural, Emergencia Climática y Transición Ecológica, Alicante, 03559 Generalitat Valenciana, Spain

**Keywords:** anticoagulant rodenticides, carcass decomposition, bromadiolone degradation, wildlife poisoning, biomonitoring

## Abstract

Bromadiolone is a second generation anticoagulant rodenticide (SGAR) used to control pest rodents worldwide. SGARs are frequently involved in secondary poisoning in rodent predators due to their persistence and toxicity. This study aims to evaluate the persistence of bromadiolone in liver at different stages of carcass decomposition in experimentally-dosed common kestrels (*Falco tinnunculus*) to understand the possibility of detecting bromadiolone in cases of wildlife poisoning and the potential risk of tertiary poisoning. Twelve individuals were divided into the bromadiolone-dose group (dosed with 55 mg/kg b.w) and the control group. Hepatic bromadiolone concentrations found in each stage of decomposition were: 3000, 2891, 4804, 4245, 8848, and 756 ng/g dry weight at 1–2 h (fresh carcass), 24 h (moderate decomposition), 72 h, 96 h (advanced decomposition), seven days (very advanced decomposition), and 15 days (initial skeletal reduction) after death, respectively. Liver bromadiolone concentrations in carcasses remained relatively stable over the first four days and raised on day 7 of decomposition under the specific conditions of this experiment, presenting a risk of causing tertiary poisoning. However, at the initial skeletal reduction stage, liver bromadiolone concentration declined, which should be considered to interpret toxicological analyses and for proper diagnosis. This experimental study provides for the first time some light to better understand the degradation of SGARs in carcasses in the wild.

## 1. Introduction

Anticoagulant rodenticides (ARs) are widely used to control pest rodents around the world [1,2,3]. They are classified into two categories according to the period in which they were developed: first generation anticoagulant rodenticides (FGARs) and second generation anticoagulant rodenticides (SGARs), the latter being more persistent and toxic after one dose [4,5]. The mechanism of action of ARs is based on the inactivation of the membrane protein vitamin K epoxide reductase in the liver, kidney, and pancreas. This inactivation leads to a reduction in vitamin K hydroquinone, which is needed for the carboxylation of clotting factors II, VII, IX, and X [6,7,8]. The reduction of blood clotting causes death by internal and external bleeding [5].

Anticoagulant rodenticides are also illegally used to kill non-target species considered harmful to agriculture, livestock-farming, and/or hunting, or as revenge between private individuals. These non-selective practices are a threat to wildlife and domestic animals [9,10,11]. This use of ARs in poisoned baits is considered an illegal action in the European Union (EU) [12,13] as well as in other countries such as the United States (US) [14], Canada [15], and in 83% of African countries [16].

The persistence and toxicity of SGARs have led to the problem of the secondary poisoning of rodent predators including mammals, scavengers, and raptors (e.g., barn owl (*Tyto alba*) and red kite (*Milvus milvus*) are frequent victims) [5,17,18,19,20,21]. These species can be exposed to low doses of ARs over multiple days, and the proportion of individuals poisoned or containing residues in their organisms has grown in the last years [5,22]. The animals exposed to sub-lethal levels of ARs over time could be weaker and more prone to infections, accidents, or predation [9,23].

Bromadiolone is the AR with the most biocidal products registered in the EU [24,25] and, together with difenacoum, the only AR used for plant protection products (PPP) [25,26]. Its use is also authorized in other countries around the world [3,27,28]. Accordingly, bromadiolone is also the most frequently detected AR in raptors worldwide [4,18,19,29,30,31,32,33,34,35,36,37]. This may be due to the fact that SGARs in general, and bromadiolone in particular, are more persistent than FGARs, having a longer half-life in the liver of prey, and thus, increased risk of secondary poisoning in predators [38]. In this regard, the bromadiolone half-life in living rats’ livers ranges from 170 to 318 days [39], while the US Environmental Protection Agency (EPA) [39] suggested that bromadiolone could persist in the liver of live rats for more than one year. However, to the best of our knowledge, data on bromadiolone persistence in decaying carcasses are lacking in the literature.

Suitable post-mortem examination and toxicological analysis are essential to deal with cases of wildlife poisoning [40]. These poisoning cases are difficult for toxicology laboratories, mainly due to the numerous products that can be involved and the variety and complexity of biological matrices with different states of decomposition. Degradation of toxic substances involved in poisoning cases in the carcass can be affected by weather conditions (e.g., sunlight, temperature, and humidity), microorganisms and cadaveric fauna leaching from the carcass to the soil, and tissue autolysis. All these factors occur during carcass decomposition and can alter the concentrations of the toxic compounds in internal tissues, which in turn will affect the correct diagnosis of wildlife poisoning. Nevertheless, few articles mention the decay status of the matrices [9,41,42] and, to the best of our knowledge, no studies have evaluated the persistence of ARs in carcasses of poisoned animals over time. Thus, it is crucial to evaluate the effect of carcass decomposition on the stability of different toxic compounds in tissues, so that an accurate interpretation of the toxicological analysis can be assured. In this sense, liver is the main metabolizing and accumulating organ for ARs, and the tissue recommended for analysis [43].

The main aim of this study was to provide a first approach to evaluate the persistence of bromadiolone over time in the liver of decomposing carcasses of experimentally-dosed common kestrels (*Falco tinnunculus*). This will improve interpretation of the presence of bromadiolone in exposed (or intoxicated) wild birds at different stages of carcass decomposition and the detection of bromadiolone in cases of wildlife poisoning as well as the risk of tertiary poisoning for scavengers.

## 2. Materials and Methods

### 2.1. Experimental Set-Up

Twelve common kestrels admitted in the “Santa Faz” Wildlife Recovery Center (WRC, Alicante, southeastern Spain) were used for the experiment. These kestrels were non-releasable and destined to be euthanized due to traumatic wing injuries preventing their release and survival in the natural environment. All individuals were physiologically healthy, with normal diet and body mass. To ensure the homogeneity of the study population, individuals were kept for at least one month under the same management conditions in proper installations at the WRC. In total, eight males and four females with body weight (b.w.) ranging from 158 to 211 g were used. Common kestrels were divided into two groups: bromadiolone-dose group (*n* = 6 individuals, four males and two females, see details below) and control group (*n* = 6 individuals, four males and two females). According to the ethics in animal experimentation, the number of animals used must be minimized, and 12 individuals were considered a sufficient number to obtain reliable data. All procedures performed complied with the ethical standards of the *Comité Ético de Experimentación Animal* (CEEA)—University of Murcia (identification code: 549/2019; date: 24 June 2019) as well as applicable institutional, local, and national guidelines and laws.

Each individual within the bromadiolone-dose group was orally dosed by providing a small piece of chicken containing the mg of the compound (bromadiolone ≥90% purchased from Sigma-Aldrich, New Haven, CT, USA). Individuals in the control group were also provided with a small piece of bromadiolone-free chicken. The LD50 for bromadiolone in the study species is not available, and inter and intraspecific differences in sensitivity to ARs have been reported [8,44]. The exact dose of bromadiolone given to each individual was 55 mg/kg b.w., half the LD50 reported for multiple bird species [4,45]. This dose was chosen to produce hepatic residues found in real cases, since raptors can be exposed to repeated sublethal doses (through rodent predation) that can be even higher than the LD50 reported for some predators [41,45,46,47].

**Figure 1:**
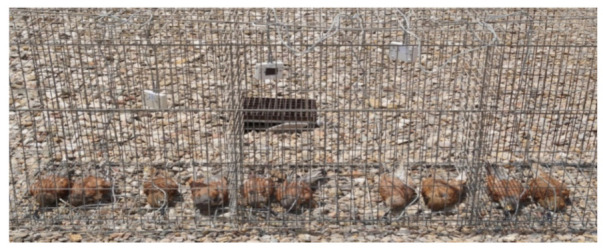
Carcasses of common kestrel (*Falco tinnunculus*) in the prone position with temperature/humidity probes.

The 12 kestrels were euthanized three days after receiving bromadiolone due to the delayed toxic action of this compound [5,48] by administering an intravenous lethal dose of sodium pentobarbital. The carcasses were immediately moved to the outdoor facilities of the Toxicology and Forensic Veterinary Service at the University of Murcia, southeast of Spain (Figure 1). The individuals were placed in a prone position, on a gravel floor, simulating a case of poisoning. They were exposed to the weather (see Table 1) 24 h a day, but were put inside a cage to avoid scavenging by large animals (Figure 1).

The decomposition experiment was performed from 4 July to 19 July 2019. The relative humidity, ambient temperature, and internal temperature of the carcasses were measured continuously using intraesophageal probes Onset TMC6-HC, and the information was recorded via Onset HOBO^®^ U12-013 dataloggers (see graph with measurements in Valverde et al. [43]). Individuals were weighed daily to evaluate the body weight loss over time. Necropsies were staggered over time (two individuals per stage, one from the bromadiolone-dose group and another one from the control group). The stages selected were: 1–2 h (day 0), 24 h (day 1), 72 h (day 3), 96 h (day 4), 7 days, and 15 days after death. During the necropsy, detailed data were recorded including date and time of necropsy, body mass measurements, sex and age of the individuals, cadaveric fauna found (e.g., eggs, larvae, insects, etc.), rigor mortis and the state of decomposition (structure, consistence, colour and other observations of eyes, tongue and oral cavity, pectoral muscle, and internal organs). Additional details can be found in a carcass decomposition protocol published elsewhere [43]. Several photographs were taken during each necropsy. Both ante-mortem and post-mortem signs related with AR intoxication were evaluated [49]. In this line, several parameters were carefully gathered after bromadiolone administration and during the necropsies including decreased mentation, weakness, pallor of mucous membranes, evidence of external (e.g., oral cavity, nares, cloaca), and/or internal haemorrhages and haematomas [49]. Samples of each organ were collected for further studies.

### 2.2. Sample Acquisition

Blood samples were collected in two stages during the experiment: (i) before bromadiolone administration to ensure that individuals did not have bromadiolone residues (in both the control and bromadiolone-dose group), and (ii) before the euthanasia (three days after bromadiolone administration) in the bromadiolone-dose group. Blood samples (ca. 2 mL) were obtained by puncturing the brachial vein with a needle (25G) and syringe and conserved in heparinized Eppendorf tubes at −20 °C until analysis.

Liver samples were taken during the necropsies and collected in polypropylene flasks and stored frozen at −20 °C until analysis. The percentage of humidity of the liver samples was calculated in an Infrared Moisture Analyzer MA35 (Sartorius) in order to indicate the results in dry weight (d.w.) and correct for different water content.

### 2.3. Chemicals and Reagents

Bromadiolone analytical standard (≥90%) was purchased from Sigma-Aldrich (New Haven, CT, USA). All solvents and reagents were of High Performance Liquid Chromatography (HPLC) quality (>99.9% purity). Acetonitrile was obtained from PanReac^®^ (Darmstadt, Germany), methanol was obtained from Lab-Scan^®^ (Gliwice, Poland), and formic acid from Probus^®^ (Badalona, Barcelona, Spain). Magnesium sulfate, sodium chloride, sodium citrate dibasic sesquihydrate, sodium citrate tribasic dihydrate, polymerically bonded, ethylenediamine-Npropyl phase that contains both primary and secondary amines (Supelclean PSA bonded silica), and C18 (Discovery DSC-18: octadecylsilane 18% C) were purchased from Supelco^®^ (Bellefonte, PA, USA).

### 2.4. Sample Preparation and Chemical Analysis

Bromadiolone was extracted from blood and liver samples using the dispersive solid phase extraction (dSPE) technique described by Gómez-Ramírez et al. [50]. Briefly, 2 g of blood or the whole homogenized liver was mixed with 2 mL of acetonitrile as the extractant. The tubes were vortexed vigorously for about a minute and a mixture of salts (1.33 g magnesium sulfate, 0.33 g sodium chloride, 0.17 g sodium citrate dibasic sesquihydrate and 0.33 g sodium citrate tribasic dehydrate) was added. The tubes were again vigorously shaken with vortex for one minute approximately. The tubes were centrifuged at 998 relative centrifugal force (RCF) for 5 min, and frozen at −20 °C for 1 h. After that, the tubes were again centrifuged in the same conditions, and the supernatant was then transferred to another tube and mixed with a new mix of salts (50 mg PSA, 50 mg DSC-18, and 300 mg magnesium sulfate). The tube was shaken and centrifuged again at 998 RCF for 5 min. The supernatant was evaporated until dry with a nitrogen stream, redissolved in 1 mL of methanol, and acidified by adding 10 µL of 5% formic acid in acetonitrile for HPLC/MS analysis.

### 2.5. Instruments and Conditions

Bromadiolone was detected and quantified using an Agilent 1290 Infinity II Series HPLC (Agilent Technologies, Santa Clara, CA, USA) equipped with an Automated Multisampler module and a High Speed Binary Pump, and connected to an Agilent 6550 Q-TOF Mass Spectrometer (Agilent Technologies, Santa Clara, CA, USA) using an Agilent Jet Stream Dual electrospray (AJS-Dual ESI) (Agilent Technologies, Santa Clara, CA, USA) interface. Experimental parameters for HPLC and Quadrupole-time-of-flight (Q-TOF) were set in MassHunter Workstation Data Acquisition software (Agilent Technologies, Rev. B.08.00).

Standards and samples (injection volume of 20 µL) were injected into a Zorbax Eclipse XDB C8, 5 µm, 150 × 4.6 mm HPLC column, at a flow rate of 0.7 mL/min. The column was thermostated at 25 °C. Solvents A (MilliQ water with 20 mM ammonium acetate) and B (methanol with 20 mM ammonium acetate) were used for the compound separation. Initial conditions were 50% solvent A and 50% solvent B. After the injection, compounds were eluted using a linear gradient 50–95% B for 22 min. Then, a linear gradient from 95–50% B was applied in 3 min and finally the system was equilibrated at starting conditions (50% B) for 10 min before a new injection.

The mass spectrometer was operated in the negative mode. The nebulizer gas pressure was set to 40 psi, whereas the drying gas flow was set to 13 L/min at a temperature of 250 °C, and the sheath gas flow was set to 12 L/min at a temperature of 300 °C. The capillary spray, nozzle, fragmentor, and octopole RF Vpp voltages were 3500 V, 1000 V, 350 V, and 750 V, respectively. Profile data in the 100–1100 m/z range were acquired for MS scans in 2 GHz extended dynamic range mode. Reference masses at 525.0707 and 586.0997 were used. The data were analyzed with MassHunter Qualitative Analysis Navigator software (version B.06.00, Service Pack 1, Agilent Technologies, Inc. US, 2012). Extracted ion chromatograms, obtained from bromadiolone molecular formula, were analyzed.

A calibration curve was prepared using two replicates of spiked chicken liver at three levels (20, 40, and 80 ng/g) and injected in HPLC/MS-TOF following the same analytical conditions as the samples. A blank containing the mobile phases A and B was injected at the beginning and at the end of the batch of samples to monitor for contamination. The same curve was used to calculate validation parameters, obtaining a correlation coefficient of *r* = 0.999 for linearity, 54.87% of recovery, and a repeatability variation coefficient of 9.59%.

### 2.6. Statistics

Statistical analyses and graphs were carried out using Microsoft Excel 2016 and SPSS v. 25. Data are presented as mean ± SD and range. Correlations between variables were tested with the Pearson correlation coefficient. Multivariate analyses of biological variables (i.e., sex coded as 1 = male and 2 =female, and body mass on day 0), weather variables (i.e., internal temperature and ambient temperature), decomposition variables (i.e., days of decomposition, body mass during necropsy, liver weight and liver water content), and bromadiolone concentration (ng/g, d.w.) were tested using principal component analysis (PCA). Tests were considered significant when *p* < 0.05.

## 3. Results and Discussion

The weather conditions during the experiment are presented in Table 1. Global mean of the period ± SD (min-max day mean) ambient air temperature, humidity, day duration, and wind speed recorded during the experiment period were 30 ± 2 (24–33) °C, 54 ± 8 (45–70)%, 14:33:45 ± 0:05:05 (14:25:00–14:41:00) hours:minutes:seconds and 9.16 ± 1.17 (6.90–11.30) km/h, respectively (detailed in Table 1).

Bromadiolone concentrations in blood and liver in both the bromadiolone-dose and the control groups are detailed in Table 2. Bromadiolone was only detected in one blood sample collected before bromadiolone administration and at low concentrations (4 ng/g wet weight (w.w.)). This suggests that common kestrels were rarely exposed to bromadiolone before the experiment. In the bromadiolone-dose group, the compound was detected in all blood samples collected three days after bromadiolone administration and before euthanasia (range: 45–135 ng/g, w.w., *n* = 6; Table 2), reflecting bromadiolone exposure and absorption due to the experimental dosing.

**Figure 2:**
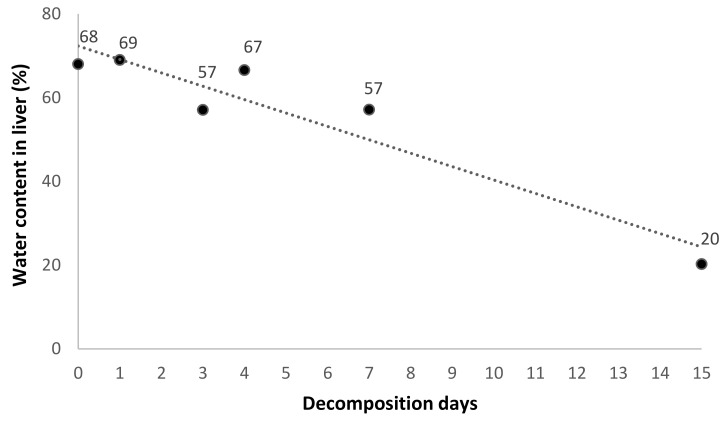
Water content in tissue (%) over time (decompositions days) in decaying carcasses of common kestrel (*r* = −0.95, *p* < 0.01). Numbers above circles indicate the mean water content (%) for the control and dosed individual at each time point.

**Figure 3:**
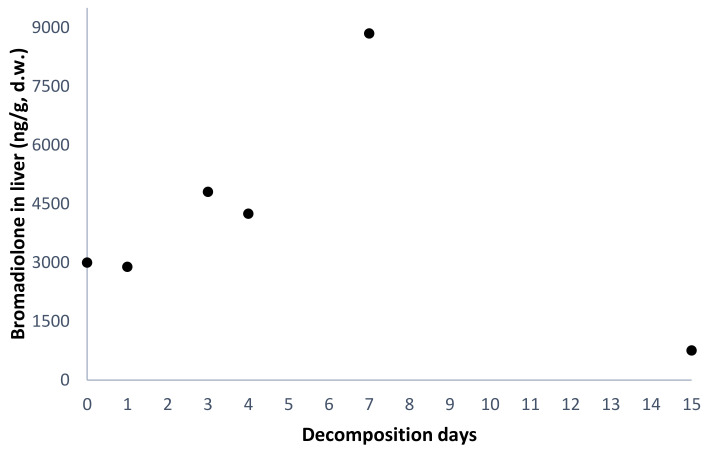
Bromadiolone concentration in liver (ng/g, d.w.) relative to the carcass decomposition time (days) in dosed common kestrel (*r* = −0.20, *p* = 0.699, *n* = 6).

Since carcasses were exposed to weather conditions, liver water content sharply decreased with time, and a negative correlation was found between tissue water content and days of decomposition (*r* = −0.95, *p* < 0.01; Figure 2). Therefore, bromadiolone concentrations are reported in both w.w. and d.w. to correct for the different water content between days (Table 2, Figure 3).

Blood is not the preferred tissue for testing AR, which raises the possibility that AR could have been present in the liver of birds before bromadiolone administration. Bromadiolone was detected at low concentrations in four control liver samples (range: 16–204 ng/g, d.w., *n* = 4; Table 2) and it was not detected in the other two control samples, which shows, in accordance with blood results, that individuals had low bromadiolone residues in the liver before the experimental dosing. Therefore, the presence of bromadiolone in the livers of the dose group before the administration cannot be discarded. However, the pre-experimental concentrations can be considered negligible compared to those in the dose group after the experimental dosing, where bromadiolone was detected in all livers (range: 756–8848 ng/g, d.w., *n* = 6). Although this was out of the scope of this article, it is important to note that free-ranging common kestrels, as rodent predators, are exposed to sublethal doses with potential health effects, particularly reproductive effects [8,51].

**Figure 4:**
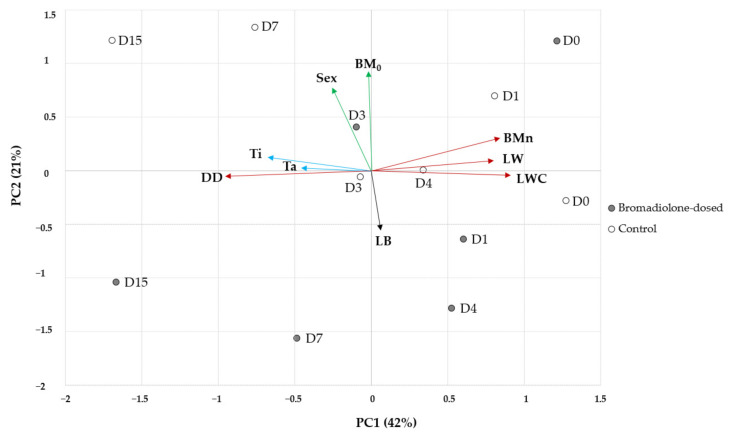
Principal component analysis (PCA) biplot of the common kestrel experiment. Vectors represent: biological variables (sex and body mass on day 0, BM_0_), weather variables (i.e., internal temperature, Ti, and ambient temperature, Ta), decomposition variables (i.e., days of decomposition, DD, body mass during necropsy, BMn, liver weight, LW, and liver water content, LWC), and liver bromadiolone concentration (LB). The points represent individual birds from the bromadiolone-dose (grey) or control group (white), and D0-D15 indicates the days of decomposition.

**Figure 5:**
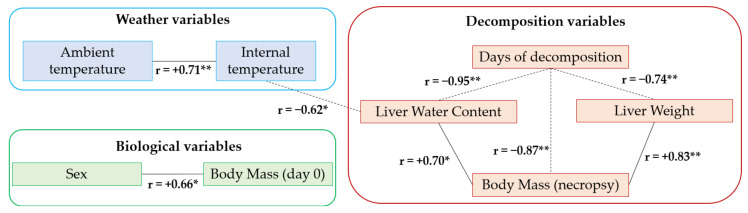
Significant relationships between weather variables (ambient and internal temperatures), biological variables (sex and body mass on day 0), and decomposition variables (days of decomposition, liver weight, liver water content and body mass during necropsy) in common kestrels. Pearson correlation coefficients (r) are presented. The directions of the relationships are shown with positive and solid lines, or negative and dashed lines. * *p* < 0.05, ** *p* ≤ 0.01.

The extracted principal components (PCs) are shown in Figure 4. Two PCs were extracted (eigenvalues: PC1 3.8 and PC2 1.9), explaining 63% of the total variation (PC1 and PC2 accounted for 42% and 21% of the variance, respectively). PC1 gave a similar weight to the variables days of decomposition (−0.95) and with opposite sign to body mass during necropsy (0.84), liver weight (0.80) and liver water content (0.91), which might be described as “decomposition” variables. PC1 also gave similar loadings to internal temperature (−0.67) and ambient temperature (−0.46), described as “weather” variables. The second component (PC2) gave more emphasis to sex (0.77) and body mass on day 0 (0.93), considered as “biological” variables, and to bromadiolone concentration (−0.53), with the opposite sign (Figure 4). In addition, the “biological” variables were significantly correlated, with females showing higher body mass, as well as the “weather” variables, with increased ambient temperatures being related to higher internal temperatures in the carcasses (Figure 5). The “decomposition” variables were also correlated. In this sense, longer decomposition time (i.e., higher days of decomposition) was related to lower body mass during necropsy, lower liver weight, and liver water content; while higher body mass during necropsy was related to higher liver weight and liver water content (Figure 5). In addition, increased internal temperatures in the carcass were related to lower liver water content (Figure 5).

PCA showed that individuals scoring highly on PC1 showed higher body mass during necropsy, liver weight and liver water content, and consequently lower days of decomposition (i.e., kestrels necropsied on days 0–1; Figure 4). Accordingly, individuals that scored highly on PC2 will have higher body mass on day 0 and lower bromadiolone concentrations in liver (Figure 4). In Figure 4, individuals in the bromadiolone-dose and control groups are indicated by different colours. In general, individuals in the bromadiolone-dose group lay below the origin and hence closer to the liver bromadiolone vector due to the higher liver bromadiolone concentrations. However, the kestrel from the bromadiolone-dose group necropsied on day 0 was positioned at the top right corner of the figure due to its highest body mass on day 0 and body mass during necropsy.

The water content in a decay sample can vary greatly depending on the decomposition stage of the carcass, which may also affect the compound concentrations (discussed below). Therefore, it is important to highlight the difficulty of comparing results between studies, since these parameters are scarcely reported. For that reason, only individuals from decomposition day 0 (fresh carcass) and day 1 (carcass at moderate decomposition stage according to the protocol developed in Valverde et al. [43]) were selected to compare concentrations with other studies in the liver.

Bromadiolone concentrations in the liver of dosed common kestrels were 960 and 896 ng/g w.w., at decomposition day 0 and 1, respectively (Table 2). No signs of AR poisoning were observed neither in the live animals after dosing nor in the necropsies (i.e., decreased mentation, weakness, pale mucous membranes, evidence of external and/or internal bleeding, hematoma [49]). This can be explained because the dose selected (half of the LD50 reported for a variety of bird species) was aimed to provide environmentally relevant doses according to concentrations reported in free-ranging wild birds. However, this lack of direct evidence should not automatically be considered as a lack of toxicity in these animals [4]. As a matter of fact, some lethal cases have been related to low AR concentrations in liver, but clinical signs compatible with AR intoxication were found, and this is considered sufficient evidence of AR-related lethal poisoning by survey networks in France and the United Kingdom [45,52]. Liver concentrations of SGARs ranging from 100 to 200 ng/g w.w. have been suggested as levels of concern in raptors, while 200 ng/g w.w. are considered critical [30,44]. However, data from secondary exposure studies show that evaluating dose-response relationships and estimating effect thresholds and tissue reference values are important challenges that need further research [8]. In biomonitoring studies in common kestrels, some liver concentrations of total ARs were above 100 ng/g w.w., with mean bromadiolone levels of 79.8 ± 34.4 ng/g w.w. in Canada [30], while in livers of common kestrels from Denmark, the median total AR (brodifacoum, bromadiolone, coumatetralyl, difenacoum, and flocoumafen) concentrations were 46 ng/g w.w., with a maximum of 679 ng/g w.w. of bromadiolone (median 0 ng/g) [30]. Bromadiolone concentrations found in liver samples in this experiment were higher than those reported in some biomonitoring studies in common kestrels [30,44]. The absence of AR intoxication signs in this study could be related to bromadiolone producing less pronounced signs of toxicity in raptors than other SGARs [8]. In addition, effect thresholds for common kestrels have not been reported, and both inter- and intraspecific variability in sensitivity to ARs have been described. In this sense, remarkable differences in AR tolerance have been described among some bird species [8].

Since bromadiolone was detected in the liver of all dosed birds, the effect of carcass degradation in bromadiolone concentrations was evaluated. Liver bromadiolone levels were not correlated with days of decomposition (*r* = −0.20, *p* = 0.699), showing that there was no progressive reduction in liver concentrations with time in this study (Figure 3). Bromadiolone concentrations found in the liver of common kestrel carcasses showed a slight (not significant) rise over the first days of decomposition, particularly evident on day 7 (Table 2, Figure 3), under the specific characteristics and weather conditions of this experiment (Table 1). On day 15, bromadiolone concentrations showed a non-significant decrease of 84% compared to the mean value observed at decomposition days 0–7 (Table 2, Figure 3). Considering that, due to ethical reasons and the availability of non-releasable individuals, the bromadiolone-dose group only had one kestrel for each decomposition stage, the individual effect was strong. Therefore, due to the limited number of samples, this trend could be a random variation in bromadiolone concentrations. However, several combined factors could partially explain these results, although further studies are needed for a proper interpretation. These factors include: (i) individual-specific condition, (ii) post-mortem drug redistribution, (iii) post-mortem tissue alteration, and (iv) bromadiolone degradation.

The post-mortem drug redistribution (PMR), in other words, movement of drugs between organs, tissues, and fluids into the body after death [53], is another factor that may influence bromadiolone concentrations in liver. The PMR occurs by different mechanisms (e.g., diffusion through blood vessels, transparietal diffusion toward the surrounding organs, bacterial activity, cell death, which produces the leakage of the substances into extracellular space, pH changes, etc.). Therefore, accurate interpretation of compound concentrations in organs can be done when a carcass is fresh, while the PMR may complicate the understanding of the results in forensic toxicology and concentrations of toxic substances in internal tissues must be carefully interpreted [53,54,55]. Moreover, there is no specific marker to evaluate how long a substance is under the effects of PMR [53,54,55]. Although PMR has been studied mainly in human medicine rather than in animals, as far as we are concerned, this forensic phenomenon also takes place in animals [53,54,55].

The post-mortem tissue alterations may also affect bromadiolone concentrations. There is a series of changes in the carcass (e.g., tissue autolysis and putrefaction) determined by different factors such as the cause of death, the size and position of the carcass, presence of cadaveric fauna or the environmental conditions (e.g., some weather conditions may favour bacterial and cadaveric fauna and the decomposition process) [56,57,58], leading to the loss of tissue integrity and mass. Liver weights of the individuals from decomposition day 0 to day 4 (including fresh, moderate, and advanced decomposition stages) ranged from 2.2 to 5.0 g in the bromadiolone-dose group. However, the liver weights of the individuals necropsied on days 7 and 15 (very advanced decomposition stage and initial skeletal reduction, respectively) were similar between them and decreased by 2.2–5.3 times the liver weights on days 0–4 (0.98 and 0.95 g, respectively). This suggests that liver tissue decreases in mass independently of the dehydration (as water content was 57 and 20%, respectively, Table 2).

Finally, on day 15, there was a non-significant drop in bromadiolone concentration that could be partially related with a degradation of the compound with time. For example, during the putrefactive processes, bacteria may produce and metabolize different compounds [54,55], which could alter bromadiolone levels. The carcass on day 15 would be less attractive for scavengers, which would mean, together with the potential bromadiolone degradation, a lower risk of tertiary poisoning for scavengers. However, this study should be considered as a first approach for future studies, but it presents some limitations regarding the number of individuals available and cannot provide clear evidence. There is a lack of literature regarding the behaviour of ARs in carcasses in the field and their potential degradation over time. Further studies including more individuals necropsied at each time point and after additional days of decomposition within this time frame (particularly from day 7 to 15) would help to properly draw the degradation curve for bromadiolone in carcasses.

## 4. Conclusions

This experimental study was limited regarding the number of individuals used at each decomposition stage due to ethical reasons and the availability of non-releasable common kestrels. Therefore, the non-significant tendency found in bromadiolone concentrations could be a random variation. However, this is the first study providing some light to better understand the degradation of SGARs in carcasses in the field. Our results could suggest that bromadiolone may persist in fresh, moderately, and advanced decomposed carcasses, although concentrations can be affected by individual-specific condition, PMR, and tissue degradation. Thus, carcasses in the field may be a source of secondary or tertiary poisoning for scavengers, at least during the first week after death when weather conditions are similar to those found in this study. However, when the carcass was at initial skeletal reduction (ca. 15 days after death), bromadiolone concentration in liver declined by 84% compared to the mean value observed at earlier decomposition stages. This result should be interpreted with caution since it represents data from a single individual. Therefore, additional research is encouraged to better interpret the degradation of the product with time. This information is essential to evaluate the risk of secondary and tertiary poisoning and for an accurate interpretation of the toxicological analysis and proper diagnosis. Considering our results, wildlife sampled 7–15 days post-mortem with low AR concentrations, but showing haemorrhaging signs, should not be immediately ruled as non-AR death due to the potential decreased trends suggested in this study.

Despite the lack of any AR intoxication sign in the experimentally-dosed common kestrels, bromadiolone levels found in liver were higher than those reported as SGARs concentrations of concern in raptors (100–200 ng/g w.w., [30]) and higher than those found in some biomonitoring studies. The absence of signs of toxicity could be due to bromadiolone producing less pronounced intoxication signs than other SGARs, and the potential inter- and intraspecific variability in sensitivity to ARs.

New experiments including more individuals necropsied after additional days of decomposition would help to properly draw the degradation curve for bromadiolone in carcasses. It is essential to undertake complementary studies on a broader variety of weather conditions and species of different sizes for a proper assessment of the persistence of bromadiolone and other ARs on wildlife carcasses. In addition, the dose-response relationships, effect thresholds, and tissue reference values should be further evaluated.

Finally, we encourage future studies to provide information on the water content and state of decomposition of samples to better evaluate concentrations and facilitate results comparison between studies. We recommend the use of protocols such as the one provided by Valverde et al. [43], which is based on a scoring method to classify stages of carcass decomposition.

## Figures and Tables

**Table 1:** Weather conditions during the experiment in common kestrel (*Falco tinnunculus*). Days after death, internal carcass temperature, ambient temperature and humidity during the experiment are provided (4–19 July 2019, sunrise 06:48–06:57 a.m. and sunset 09:31–09:26 p.m., raining 0 mm, mean wind speed 9.16 km/h, Murcia, Spain).

Days after Death	Internal Temperature (°C) ^1^	Ambient Temperature (°C) ^1^	Relative Humidity (%) ^1^
Day 0	NM	NM	NM
Day 1	32.87	30.17	58.31
Day 3	32.74	33.17	44.63
Day 4	28.67	27.97	60.33
Day 7	32.81	30.10	50.65
Day 15	33.90	30.09	NM

^1^ The mean value for all individuals per day is presented. NM: Not measured.

**Table 2:** Bromadiolone concentration in blood (before dosing and euthanasia) and liver (according to the days of decomposition) of common kestrels in the bromadiolone-dose and control groups.

ID	Sex	Group	Blood		Liver			
			Concentration before Dosing (ng/g, w.w.)	Concentration before Euthanasia (ng/g, w.w.)	Decomposition Day	Liver Weight (g)	Concentration (ng/g, w.w.)	Concentration (ng/g, d.w.)
#1	F	Bromadiolone-dose group	nd	47	0	5.0	960	3000
#3	M		nd	57	1	4.1	896	2891
#5	F		nd	45	3	2.2	2062	4804
#7	M		4	135	4	2.2	1419	4245
#9	M		nd	76	7	1.0	3794	8848
#11	M		nd	60	15	1.0	603	756
#2	M	Control group	nd	NA	0	6.0	65	204
#4	M		nd	NA	1	5.2	12	38
#6	M		nd	NA	3	2.8	36	84
#8	M		nd	NA	4	0.5	nd	nd
#10	F		nd	NA	7	0.5	nd	nd
#12	F		nd	NA	15	0.7	13	16

M: male, F: female, w.w.: wet weight; d.w.: dry weight; nd: not detected, NA: not analyzed.
